# 3-Cyclo­pentyl­sulfonyl-5-fluoro-2-methyl-1-benzofuran

**DOI:** 10.1107/S1600536811021222

**Published:** 2011-06-18

**Authors:** Pil Ja Seo, Hong Dae Choi, Byeng Wha Son, Uk Lee

**Affiliations:** a Department of Chemistry, Dongeui University, San 24 Kaya-dong Busanjin-gu, Busan 614-714, Republic of Korea; b Department of Chemistry, Pukyong National University, 599-1 Daeyeon 3-dong, Nam-gu, Busan 608-737, Republic of Korea

## Abstract

There are two independent mol­ecules, *A* and *B*, in the asymmetric unit of the title compound, C_14_H_15_FO_3_S, in each of which the cyclo­pentyl ring adopts an envelope conformation. The benzofuran units in each mol­ecule are essentially planar, with mean deviations from the least-squares plane defined by the nine constituent ring atoms of 0.009 (2) Å for mol­ecule *A* and 0.013 (2) Å for mol­ecule *B*. In the crystal, mol­ecules are linked by weak C—H⋯O hydrogen bonds. In the cyclo­pentyl ring of mol­ecule *B*, one C atom is disordered over two positions with site-occupancy factors of 0.60 (2) and 0.40 (2).

## Related literature

For the pharmacological activity of benzofuran compounds, see: Aslam *et al.* (2009 ▶); Galal *et al.* (2009 ▶); Khan *et al.* (2005 ▶). For natural products with benzofuran rings, see: Akgul & Anil (2003 ▶); Soekamto *et al.* (2003 ▶). For a structural study of the related compound 5-bromo-3-cyclo­pentyl­sulfinyl-2-methyl-1-benzofuran, see: Seo *et al.* (2011 ▶).
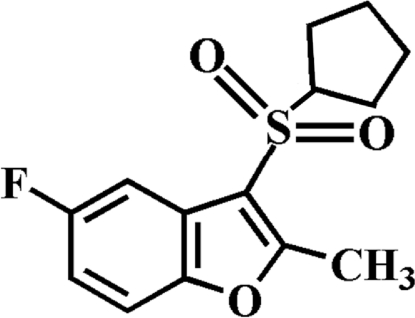


## Experimental

### 

#### Crystal data

C_14_H_15_FO_3_S
*M* *_r_* = 282.32
Triclinic, 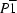

*a* = 10.0568 (8) Å
*b* = 10.2697 (8) Å
*c* = 13.2894 (10) Å
α = 95.033 (4)°
β = 109.140 (4)°
γ = 91.229 (4)°
*V* = 1289.82 (17) Å^3^
*Z* = 4
Mo *K*α radiation
μ = 0.26 mm^−1^
*T* = 173 K
0.26 × 0.24 × 0.20 mm

#### Data collection

Bruker SMART APEXII CCD diffractometer
Absorption correction: multi-scan (*SADABS*; Bruker, 2009 ▶) *T*_min_ = 0.936, *T*_max_ = 0.948
21777 measured reflections
5565 independent reflections
4022 reflections with *I* > 2σ(*I*)
*R*_int_ = 0.042

#### Refinement

*R*[*F* ^2^ > 2σ(*F* ^2^)] = 0.045
*wR*(*F* ^2^) = 0.119
*S* = 1.03
5565 reflections
361 parameters
20 restraints
H atoms treated by a mixture of independent and constrained refinement
Δρ_max_ = 0.50 e Å^−3^
Δρ_min_ = −0.31 e Å^−3^

### 

Data collection: *APEX2* (Bruker, 2009 ▶); cell refinement: *SAINT* (Bruker, 2009 ▶); data reduction: *SAINT*; program(s) used to solve structure: *SHELXS97* (Sheldrick, 2008 ▶); program(s) used to refine structure: *SHELXL97* (Sheldrick, 2008 ▶); molecular graphics: *ORTEP-3* (Farrugia, 1997 ▶) and *DIAMOND* (Brandenburg, 1998 ▶); software used to prepare material for publication: *SHELXL97*.

## Supplementary Material

Crystal structure: contains datablock(s) global, I. DOI: 10.1107/S1600536811021222/go2016sup1.cif

Structure factors: contains datablock(s) I. DOI: 10.1107/S1600536811021222/go2016Isup2.hkl

Supplementary material file. DOI: 10.1107/S1600536811021222/go2016Isup3.cml

Additional supplementary materials:  crystallographic information; 3D view; checkCIF report

## Figures and Tables

**Table 1:** Hydrogen-bond geometry (Å, °)

*D*—H⋯*A*	*D*—H	H⋯*A*	*D*⋯*A*	*D*—H⋯*A*
C9—H9*C*⋯O6^i^	0.98	2.45	3.348 (3)	152
C19—H19⋯O6^ii^	0.95	2.58	3.469 (3)	157
C23—H23*A*⋯O2^iii^	0.98	2.50	3.318 (3)	141

Symmetry codes: (i) 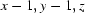; (ii) 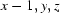; (iii) 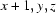.

**Fig. 1.**
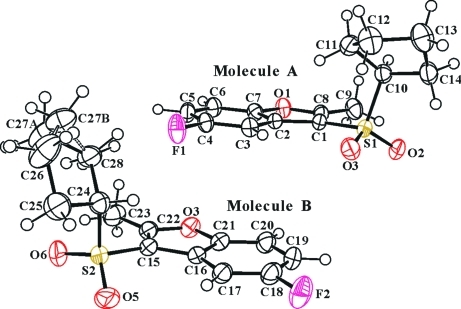
The molecular structure of the title compound with the atom numbering scheme. Displacement ellipsoids are drawn at the 50% probability level. H atoms are presented as a small spheres of arbitrary radius.

**Fig. 2.**
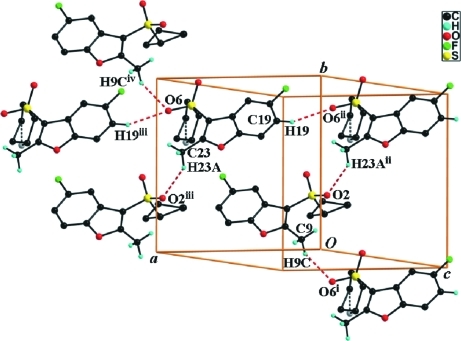
A view of the C–H···O interactions (dotted lines) in the crystal structure of the title compound. [Symmetry codes: (i) x - 1, y - 1, z; (ii) x - 1, y, z; (iii) x + 1, y, z; (iv) x + 1, y + 1, z.]

## supplementary crystallographic information

### Comment

Many compounds having a benzofuran moiety have attracted much attention due to their valuable biological properties such as antifungal, antimicrobial, antitumor and antiviral activities (Aslam *et al.*, 2009; Galal *et al.*, 2009; Khan *et al.*, 2005). These benzofuran derivatives occur in a wide range of natural products (Akgul & Anil, 2003; Soekamto *et al.*, 2003). As a part of our ongoing studies of the substituent effect on the solid state structures of 3-cyclopentylsulfinyl-5-halo-2-methyl-1-benzofuran analogues (Seo *et al.*, 2011), we report herein on the crystal structure of the title compound.

The asymmetric unit of the title compound is shown in Fig. 1. There are two independent unique molecules [labelled A & B] in which the benzofuran unit is essentially planar, with a mean deviation of 0.009 (2) Å for A and 0.013 (2) Å for B, respectively, from the least-squares plane defined by the nine constituent atoms. The cyclopentyl rings of both molecules are in an envelope form. In the cyclopentyl ring of molecule B, C27 atom is disordered over two positions with site-occupancy factors, from refinement of 0.60 (2) (part 1) and 0.40 (8) (part 2). In the crystal packing (Fig. 2), molecules are linked by weak non-classical intermolecular C–H···O hydrogen bonds; the first one between a methyl H atom and the O atom of the sulfonyl group (Table 1; C9–H9C···O6^i^), the second one between a benzene H atom and the O atom of the sulfonyl group (Table 1; C19–H19···O6^ii^), and the third one between a methyl H atom and the O atom of the sulfonyl group (Table 1; C23–H23A···O2^iii^).

### Experimental

3-chloroperoxybenzoic acid (77%, 560 mg, 2.5 mmol) was added in small portions to a stirred solution of 3-cyclopentylsulfanyl-5-fluoro-2-methyl-1-benzofuran (325 mg, 1.2 mmol) in dichloromethane (40 mL) at 273 K. After being stirred at room temperature for 8h, the mixture was washed with saturated sodium bicarbonate solution and the organic layer was separated, dried over magnesium sulfate, filtered and concentrated at reduced pressure. The residue was purified by column chromatography (hexane-ethyl acetate, 4:1 v/v) to afford the title compound as a colorless solid [yield 71%, m.p. 394-395 K; *R*_f_ = 0.49 (hexane-ethyl acetate, 4:1 v/v)]. Single crystals suitable for X-ray diffraction were prepared by slow evaporation of a solution of the title compound in ethyl acetate at room temperature.

### Refinement

All H atoms were positioned geometrically and refined using a riding model, with C–H = 0.95 Å for aryl, 1.00 Å for methine, 0.99 Å for methylene and 0.98 Å for methyl H atoms, respectively. *U*_iso_(H) = 1.2*U*_eq_(C) for aryl, methine, methylene, and 1.5*U*_eq_(C) for methyl H atoms. The C27 atom of the cyclopentyl ring is disordered over two positions with site-ccupancy factors, from refinement of 0.60 (2) (part A) and 0.40 (2) (part B). The C–C distance sets were restrained to be with 0.003 Å using command DFIX and SADI, and thermal ellipsoid parameters of C27A and C27B set were restrained to 0.01 using commend ISOR.

### Crystal data

**Table:**

C_14_H_15_FO_3_S	*Z* = 4
*M**_r_* = 282.32	*F*(000) = 592
Triclinic, *P*1	*D*_x_ = 1.454 Mg m^−^^3^
Hall symbol: -P 1	Melting point = 394–395 K
*a* = 10.0568 (8) Å	Mo *K*α radiation, λ = 0.71073 Å
*b* = 10.2697 (8) Å	Cell parameters from 6383 reflections
*c* = 13.2894 (10) Å	θ = 2.2–28.1°
α = 95.033 (4)°	µ = 0.26 mm^−^^1^
β = 109.140 (4)°	*T* = 173 K
γ = 91.229 (4)°	Block, colourless
*V* = 1289.82 (17) Å^3^	0.26 × 0.24 × 0.20 mm

### Data collection

**Table:**

Bruker SMART APEXII CCD diffractometer	5565 independent reflections
Radiation source: rotating anode	4022 reflections with *I* > 2σ(*I*)
graphite multilayer	*R*_int_ = 0.042
Detector resolution: 10.0 pixels mm^-1^	θ_max_ = 27.0°, θ_min_ = 1.6°
φ and ω scans	*h* = −12→11
Absorption correction: multi-scan (*SADABS*; Bruker, 2009)	*k* = −13→13
*T*_min_ = 0.936, *T*_max_ = 0.948	*l* = −16→16
21777 measured reflections	

### Refinement

**Table:**

Refinement on *F*^2^	Primary atom site location: structure-invariant direct methods
Least-squares matrix: full	Secondary atom site location: difference Fourier map
*R*[*F*^2^ > 2σ(*F*^2^)] = 0.045	Hydrogen site location: difference Fourier map
*wR*(*F*^2^) = 0.119	H atoms treated by a mixture of independent and constrained refinement
*S* = 1.03	*w* = 1/[σ^2^(*F*_o_^2^) + (0.0514*P*)^2^ + 0.6137*P*] where *P* = (*F*_o_^2^ + 2*F*_c_^2^)/3
5565 reflections	(Δ/σ)_max_ = 0.001
361 parameters	Δρ_max_ = 0.50 e Å^−^^3^
20 restraints	Δρ_min_ = −0.31 e Å^−^^3^

### Special details

**Table:**

Geometry. All esds (except the esd in the dihedral angle between two l.s. planes) are estimated using the full covariance matrix. The cell esds are taken into account individually in the estimation of esds in distances, angles and torsion angles; correlations between esds in cell parameters are only used when they are defined by crystal symmetry. An approximate (isotropic) treatment of cell esds is used for estimating esds involving l.s. planes.
Refinement. Refinement of F^2^ against ALL reflections. The weighted R-factor wR and goodness of fit S are based on F^2^, conventional R-factors R are based on F, with F set to zero for negative F^2^. The threshold expression of F^2^ > 2sigma(F^2^) is used only for calculating R-factors(gt) etc. and is not relevant to the choice of reflections for refinement. R-factors based on F^2^ are statistically about twice as large as those based on F, and R- factors based on ALL data will be even larger.

### Fractional atomic coordinates and isotropic or equivalent isotropic displacement parameters (Å^2^)

**Table:**

	*x*	*y*	*z*	*U*_iso_*/*U*_eq_	Occ. (<1)
S1	0.23453 (6)	0.33785 (5)	0.23641 (4)	0.03150 (15)	
F1	0.81952 (14)	0.46170 (14)	0.28853 (11)	0.0484 (4)	
O1	0.40319 (15)	0.10269 (14)	0.07061 (11)	0.0326 (4)	
O2	0.09116 (17)	0.32983 (18)	0.16639 (13)	0.0460 (4)	
C1	0.3369 (2)	0.2567 (2)	0.17088 (16)	0.0269 (4)	
C2	0.4835 (2)	0.28575 (19)	0.18530 (15)	0.0252 (4)	
O3	0.30185 (18)	0.46418 (15)	0.27859 (14)	0.0438 (4)	
C3	0.5859 (2)	0.3822 (2)	0.24350 (16)	0.0296 (5)	
H3	0.5664	0.4524	0.2872	0.036*	
C4	0.7165 (2)	0.3693 (2)	0.23369 (17)	0.0311 (5)	
C5	0.7522 (2)	0.2698 (2)	0.17204 (18)	0.0344 (5)	
H5	0.8456	0.2666	0.1699	0.041*	
C6	0.6504 (2)	0.1750 (2)	0.11357 (17)	0.0333 (5)	
H6	0.6704	0.1051	0.0699	0.040*	
C7	0.5185 (2)	0.1872 (2)	0.12172 (16)	0.0280 (5)	
C8	0.2955 (2)	0.1465 (2)	0.10210 (16)	0.0305 (5)	
C9	0.1621 (2)	0.0657 (2)	0.05858 (19)	0.0411 (6)	
H9A	0.1435	0.0392	−0.0177	0.062*	
H9B	0.0847	0.1166	0.0675	0.062*	
H9C	0.1697	−0.0125	0.0971	0.062*	
C10	0.2422 (2)	0.2431 (2)	0.34337 (17)	0.0310 (5)	
H10	0.2079	0.1510	0.3140	0.037*	
C11	0.3910 (3)	0.2436 (2)	0.42411 (18)	0.0396 (6)	
H11A	0.4133	0.1548	0.4460	0.048*	
H11B	0.4617	0.2748	0.3933	0.048*	
C12	0.3888 (3)	0.3373 (3)	0.5190 (2)	0.0514 (7)	
H12A	0.4612	0.3171	0.5860	0.062*	
H12B	0.4047	0.4293	0.5070	0.062*	
C13	0.2438 (3)	0.3130 (3)	0.5228 (2)	0.0510 (7)	
H13A	0.2174	0.3868	0.5643	0.061*	
H13B	0.2374	0.2315	0.5560	0.061*	
C14	0.1491 (3)	0.3006 (2)	0.40645 (19)	0.0405 (6)	
H14A	0.1171	0.3872	0.3839	0.049*	
H14B	0.0654	0.2414	0.3954	0.049*	
S2	0.97507 (6)	0.86902 (6)	0.24244 (5)	0.03691 (16)	
F2	0.43771 (16)	0.96231 (15)	0.28861 (13)	0.0589 (4)	
O4	0.67248 (16)	0.60770 (14)	0.08407 (12)	0.0359 (4)	
O5	0.93729 (18)	1.00229 (16)	0.24654 (16)	0.0536 (5)	
O6	1.07814 (18)	0.83460 (19)	0.19339 (15)	0.0534 (5)	
C15	0.8217 (2)	0.7728 (2)	0.17896 (17)	0.0308 (5)	
C16	0.6865 (2)	0.7948 (2)	0.19121 (16)	0.0290 (5)	
C17	0.6327 (2)	0.8908 (2)	0.24526 (17)	0.0332 (5)	
H17	0.6884	0.9657	0.2853	0.040*	
C18	0.4942 (3)	0.8703 (2)	0.23702 (19)	0.0399 (6)	
C19	0.4080 (3)	0.7648 (2)	0.1794 (2)	0.0424 (6)	
H19	0.3129	0.7566	0.1776	0.051*	
C20	0.4606 (2)	0.6713 (2)	0.12428 (19)	0.0387 (5)	
H20	0.4039	0.5976	0.0830	0.046*	
C21	0.5994 (2)	0.6900 (2)	0.13206 (17)	0.0312 (5)	
C22	0.8073 (2)	0.6592 (2)	0.11486 (17)	0.0334 (5)	
C23	0.9032 (3)	0.5815 (3)	0.0738 (2)	0.0456 (6)	
H23A	0.9124	0.4962	0.1026	0.068*	
H23B	0.9960	0.6279	0.0961	0.068*	
H23C	0.8650	0.5686	−0.0045	0.068*	
C24	1.0323 (2)	0.8246 (2)	0.37518 (18)	0.0367 (5)	
H24	0.9527	0.8329	0.4044	0.044*	
C25	1.1571 (3)	0.9139 (3)	0.4469 (2)	0.0526 (7)	
H25A	1.2183	0.9408	0.4064	0.063*	
H25B	1.1246	0.9932	0.4779	0.063*	
C26	1.2325 (3)	0.8310 (2)	0.5315 (2)	0.0665 (9)	
H26A	1.1804	0.8185	0.5819	0.080*	0.60 (2)
H26B	1.3293	0.8672	0.5719	0.080*	0.60 (2)
H26C	1.1987	0.8460	0.5934	0.080*	0.40 (2)
H26D	1.3349	0.8542	0.5562	0.080*	0.40 (2)
C27A	1.2327 (6)	0.7051 (8)	0.4620 (8)	0.060 (3)	0.60 (2)
H27A	1.3006	0.7139	0.4230	0.072*	0.60 (2)
H27B	1.2574	0.6306	0.5058	0.072*	0.60 (2)
C27B	1.1998 (14)	0.6866 (3)	0.4914 (7)	0.050 (3)	0.40 (2)
H27C	1.2837	0.6453	0.4824	0.060*	0.40 (2)
H27D	1.1679	0.6392	0.5419	0.060*	0.40 (2)
C28	1.0829 (3)	0.6861 (2)	0.3846 (2)	0.0429 (6)	
H28A	1.0237	0.6318	0.4132	0.051*	0.60 (2)
H28B	1.0810	0.6439	0.3142	0.051*	0.60 (2)
H28C	1.012 (7)	0.612 (7)	0.354 (5)	0.051*	0.40 (2)
H28D	1.134 (8)	0.675 (7)	0.343 (5)	0.051*	0.40 (2)

### Atomic displacement parameters (Å^2^)

**Table:**

	*U*^11^	*U*^22^	*U*^33^	*U*^12^	*U*^13^	*U*^23^
S1	0.0255 (3)	0.0333 (3)	0.0406 (3)	0.0073 (2)	0.0156 (2)	0.0095 (2)
F1	0.0306 (8)	0.0513 (8)	0.0585 (9)	−0.0166 (6)	0.0147 (7)	−0.0158 (7)
O1	0.0280 (8)	0.0345 (8)	0.0335 (8)	−0.0066 (6)	0.0106 (7)	−0.0059 (6)
O2	0.0253 (9)	0.0664 (12)	0.0505 (10)	0.0139 (8)	0.0136 (8)	0.0204 (9)
C1	0.0210 (10)	0.0323 (11)	0.0289 (11)	0.0036 (8)	0.0093 (8)	0.0064 (9)
C2	0.0241 (11)	0.0269 (10)	0.0266 (10)	0.0031 (8)	0.0103 (8)	0.0048 (8)
O3	0.0478 (10)	0.0284 (8)	0.0672 (11)	0.0050 (7)	0.0350 (9)	0.0058 (8)
C3	0.0292 (12)	0.0291 (11)	0.0305 (11)	−0.0005 (9)	0.0112 (9)	−0.0016 (9)
C4	0.0240 (11)	0.0318 (11)	0.0347 (11)	−0.0067 (9)	0.0073 (9)	−0.0002 (9)
C5	0.0247 (11)	0.0387 (12)	0.0418 (13)	0.0011 (9)	0.0139 (10)	0.0036 (10)
C6	0.0328 (12)	0.0321 (11)	0.0370 (12)	0.0025 (9)	0.0160 (10)	−0.0029 (9)
C7	0.0267 (11)	0.0287 (11)	0.0272 (10)	−0.0035 (9)	0.0079 (9)	0.0003 (8)
C8	0.0237 (11)	0.0380 (12)	0.0295 (11)	−0.0011 (9)	0.0079 (9)	0.0050 (9)
C9	0.0302 (13)	0.0490 (14)	0.0402 (13)	−0.0123 (11)	0.0086 (10)	−0.0014 (11)
C10	0.0313 (12)	0.0297 (11)	0.0353 (11)	0.0024 (9)	0.0153 (9)	0.0035 (9)
C11	0.0370 (13)	0.0476 (14)	0.0365 (12)	0.0095 (11)	0.0137 (10)	0.0082 (10)
C12	0.0499 (16)	0.0596 (17)	0.0428 (14)	−0.0057 (13)	0.0161 (12)	−0.0057 (12)
C13	0.0549 (17)	0.0577 (17)	0.0448 (15)	−0.0044 (13)	0.0266 (13)	−0.0091 (12)
C14	0.0375 (14)	0.0428 (13)	0.0483 (14)	0.0030 (11)	0.0240 (11)	0.0039 (11)
S2	0.0264 (3)	0.0356 (3)	0.0497 (4)	0.0012 (2)	0.0113 (3)	0.0147 (3)
F2	0.0464 (9)	0.0605 (10)	0.0742 (11)	0.0162 (8)	0.0294 (8)	−0.0092 (8)
O4	0.0350 (9)	0.0342 (8)	0.0360 (8)	0.0045 (7)	0.0096 (7)	−0.0021 (7)
O5	0.0420 (10)	0.0311 (9)	0.0794 (13)	−0.0014 (8)	0.0065 (9)	0.0158 (9)
O6	0.0326 (10)	0.0718 (13)	0.0651 (12)	0.0029 (9)	0.0248 (9)	0.0214 (10)
C15	0.0278 (12)	0.0328 (11)	0.0347 (11)	0.0066 (9)	0.0120 (9)	0.0098 (9)
C16	0.0262 (11)	0.0308 (11)	0.0301 (11)	0.0035 (9)	0.0081 (9)	0.0080 (9)
C17	0.0300 (12)	0.0311 (11)	0.0375 (12)	0.0031 (9)	0.0099 (10)	0.0024 (9)
C18	0.0388 (14)	0.0397 (13)	0.0444 (14)	0.0123 (11)	0.0176 (11)	0.0041 (11)
C19	0.0253 (12)	0.0509 (15)	0.0521 (15)	0.0053 (11)	0.0129 (11)	0.0109 (12)
C20	0.0291 (12)	0.0377 (13)	0.0443 (13)	−0.0010 (10)	0.0059 (10)	0.0023 (10)
C21	0.0307 (12)	0.0299 (11)	0.0325 (11)	0.0059 (9)	0.0095 (9)	0.0034 (9)
C22	0.0327 (12)	0.0372 (12)	0.0340 (12)	0.0086 (10)	0.0139 (10)	0.0102 (9)
C23	0.0499 (16)	0.0497 (15)	0.0450 (14)	0.0195 (12)	0.0246 (12)	0.0076 (11)
C24	0.0318 (12)	0.0339 (12)	0.0417 (13)	−0.0020 (10)	0.0091 (10)	0.0031 (10)
C25	0.0480 (16)	0.0382 (14)	0.0609 (17)	−0.0087 (12)	0.0060 (13)	−0.0013 (12)
C26	0.0509 (18)	0.0546 (18)	0.069 (2)	0.0086 (14)	−0.0089 (15)	−0.0162 (15)
C27A	0.046 (3)	0.050 (3)	0.058 (4)	0.016 (3)	−0.013 (3)	−0.009 (3)
C27B	0.059 (6)	0.040 (4)	0.042 (4)	0.011 (4)	0.005 (4)	0.000 (3)
C28	0.0407 (15)	0.0318 (13)	0.0478 (15)	−0.0022 (11)	0.0034 (12)	0.0050 (11)

### Geometric parameters (Å, °)

**Table:**

S1—O3	1.4289 (17)	F2—C18	1.360 (3)
S1—O2	1.4329 (17)	O4—C22	1.362 (3)
S1—C1	1.735 (2)	O4—C21	1.379 (3)
S1—C10	1.774 (2)	C15—C22	1.358 (3)
F1—C4	1.359 (2)	C15—C16	1.442 (3)
O1—C8	1.355 (3)	C16—C21	1.380 (3)
O1—C7	1.377 (2)	C16—C17	1.390 (3)
C1—C8	1.356 (3)	C17—C18	1.372 (3)
C1—C2	1.444 (3)	C17—H17	0.9500
C2—C7	1.387 (3)	C18—C19	1.375 (3)
C2—C3	1.392 (3)	C19—C20	1.375 (3)
C3—C4	1.370 (3)	C19—H19	0.9500
C3—H3	0.9500	C20—C21	1.374 (3)
C4—C5	1.381 (3)	C20—H20	0.9500
C5—C6	1.379 (3)	C22—C23	1.471 (3)
C5—H5	0.9500	C23—H23A	0.9800
C6—C7	1.373 (3)	C23—H23B	0.9800
C6—H6	0.9500	C23—H23C	0.9800
C8—C9	1.478 (3)	C24—C28	1.523 (3)
C9—H9A	0.9800	C24—C25	1.529 (3)
C9—H9B	0.9800	C24—H24	1.0000
C9—H9C	0.9800	C25—C26	1.484 (4)
C10—C11	1.528 (3)	C25—H25A	0.9900
C10—C14	1.543 (3)	C25—H25B	0.9900
C10—H10	1.0000	C26—C27B	1.522 (2)
C11—C12	1.524 (3)	C26—C27A	1.522 (2)
C11—H11A	0.9900	C26—H26A	0.9900
C11—H11B	0.9900	C26—H26B	0.9900
C12—C13	1.492 (4)	C26—H26C	0.9900
C12—H12A	0.9900	C26—H26D	0.9900
C12—H12B	0.9900	C27A—C28	1.5166 (18)
C13—C14	1.520 (3)	C27A—H27A	0.9900
C13—H13A	0.9900	C27A—H27B	0.9900
C13—H13B	0.9900	C27B—C28	1.5171 (18)
C14—H14A	0.9900	C27B—H27C	0.9900
C14—H14B	0.9900	C27B—H27D	0.9900
S2—O6	1.4292 (19)	C28—H28A	0.9900
S2—O5	1.4294 (18)	C28—H28B	0.9900
S2—C15	1.733 (2)	C28—H28C	0.99 (7)
S2—C24	1.771 (2)	C28—H28D	0.87 (7)
			
O3—S1—O2	118.65 (11)	F2—C18—C19	117.8 (2)
O3—S1—C1	107.46 (10)	C17—C18—C19	124.8 (2)
O2—S1—C1	108.81 (10)	C20—C19—C18	119.4 (2)
O3—S1—C10	109.46 (10)	C20—C19—H19	120.3
O2—S1—C10	107.34 (10)	C18—C19—H19	120.3
C1—S1—C10	104.18 (10)	C21—C20—C19	116.5 (2)
C8—O1—C7	107.10 (15)	C21—C20—H20	121.7
C8—C1—C2	107.13 (19)	C19—C20—H20	121.7
C8—C1—S1	125.44 (16)	C20—C21—O4	125.7 (2)
C2—C1—S1	127.23 (16)	C20—C21—C16	124.1 (2)
C7—C2—C3	119.14 (19)	O4—C21—C16	110.18 (19)
C7—C2—C1	104.77 (17)	C15—C22—O4	110.5 (2)
C3—C2—C1	136.1 (2)	C15—C22—C23	134.7 (2)
C4—C3—C2	115.69 (19)	O4—C22—C23	114.8 (2)
C4—C3—H3	122.2	C22—C23—H23A	109.5
C2—C3—H3	122.2	C22—C23—H23B	109.5
F1—C4—C3	117.72 (19)	H23A—C23—H23B	109.5
F1—C4—C5	117.06 (19)	C22—C23—H23C	109.5
C3—C4—C5	125.22 (19)	H23A—C23—H23C	109.5
C6—C5—C4	119.1 (2)	H23B—C23—H23C	109.5
C6—C5—H5	120.5	C28—C24—C25	105.42 (19)
C4—C5—H5	120.5	C28—C24—S2	113.88 (17)
C7—C6—C5	116.5 (2)	C25—C24—S2	111.11 (17)
C7—C6—H6	121.8	C28—C24—H24	108.8
C5—C6—H6	121.8	C25—C24—H24	108.8
C6—C7—O1	125.45 (19)	S2—C24—H24	108.8
C6—C7—C2	124.39 (19)	C26—C25—C24	103.5 (2)
O1—C7—C2	110.14 (18)	C26—C25—H25A	111.1
O1—C8—C1	110.84 (17)	C24—C25—H25A	111.1
O1—C8—C9	115.52 (19)	C26—C25—H25B	111.1
C1—C8—C9	133.6 (2)	C24—C25—H25B	111.1
C8—C9—H9A	109.5	H25A—C25—H25B	109.0
C8—C9—H9B	109.5	C25—C26—C27B	110.6 (2)
H9A—C9—H9B	109.5	C25—C26—C27A	99.5 (5)
C8—C9—H9C	109.5	C25—C26—H26A	111.9
H9A—C9—H9C	109.5	C27B—C26—H26A	88.1
H9B—C9—H9C	109.5	C27A—C26—H26A	111.9
C11—C10—C14	106.14 (18)	C25—C26—H26B	111.9
C11—C10—S1	112.41 (15)	C27B—C26—H26B	122.4
C14—C10—S1	110.05 (16)	C27A—C26—H26B	111.9
C11—C10—H10	109.4	H26A—C26—H26B	109.6
C14—C10—H10	109.4	C25—C26—H26C	109.7
S1—C10—H10	109.4	C27B—C26—H26C	106.2
C12—C11—C10	104.7 (2)	C27A—C26—H26C	129.9
C12—C11—H11A	110.8	H26B—C26—H26C	94.1
C10—C11—H11A	110.8	C25—C26—H26D	110.0
C12—C11—H11B	110.8	C27B—C26—H26D	111.9
C10—C11—H11B	110.8	C27A—C26—H26D	98.1
H11A—C11—H11B	108.9	H26A—C26—H26D	122.4
C13—C12—C11	103.0 (2)	H26C—C26—H26D	108.3
C13—C12—H12A	111.2	C28—C27A—C26	104.3 (2)
C11—C12—H12A	111.2	C28—C27A—H27A	110.9
C13—C12—H12B	111.2	C26—C27A—H27A	110.9
C11—C12—H12B	111.2	C28—C27A—H27B	110.9
H12A—C12—H12B	109.1	C26—C27A—H27B	110.9
C12—C13—C14	104.7 (2)	H27A—C27A—H27B	108.9
C12—C13—H13A	110.8	C26—C27A—H28D	124 (3)
C14—C13—H13A	110.8	H27A—C27A—H28D	78.9
C12—C13—H13B	110.8	H27B—C27A—H28D	117.5
C14—C13—H13B	110.8	C28—C27B—C26	104.3 (2)
H13A—C13—H13B	108.9	C28—C27B—H27C	110.9
C13—C14—C10	104.66 (19)	C26—C27B—H27C	110.9
C13—C14—H14A	110.8	C28—C27B—H27D	110.9
C10—C14—H14A	110.8	C26—C27B—H27D	110.9
C13—C14—H14B	110.8	H27C—C27B—H27D	108.9
C10—C14—H14B	110.8	C27A—C28—C24	103.9 (3)
H14A—C14—H14B	108.9	C27B—C28—C24	107.6 (3)
O6—S2—O5	118.23 (11)	C27A—C28—H28A	111.0
O6—S2—C15	109.20 (11)	C27B—C28—H28A	88.1
O5—S2—C15	107.56 (10)	C24—C28—H28A	111.0
O6—S2—C24	109.04 (11)	C27A—C28—H28B	111.0
O5—S2—C24	107.64 (11)	C27B—C28—H28B	127.7
C15—S2—C24	104.28 (10)	C24—C28—H28B	111.0
C22—O4—C21	107.03 (16)	H28A—C28—H28B	109.0
C22—C15—C16	107.17 (19)	C27A—C28—H28C	137 (4)
C22—C15—S2	127.18 (18)	C27B—C28—H28C	123 (4)
C16—C15—S2	125.52 (17)	C24—C28—H28C	118 (4)
C21—C16—C17	119.4 (2)	H28A—C28—H28C	45.4
C21—C16—C15	105.14 (19)	H28B—C28—H28C	64.8
C17—C16—C15	135.4 (2)	C27A—C28—H28D	76 (4)
C18—C17—C16	115.7 (2)	C27B—C28—H28D	98 (5)
C18—C17—H17	122.2	C24—C28—H28D	107 (5)
C16—C17—H17	122.2	H28A—C28—H28D	137.9
F2—C18—C17	117.4 (2)	H28C—C28—H28D	100 (6)
			
O3—S1—C1—C8	−165.58 (19)	C24—S2—C15—C16	76.3 (2)
O2—S1—C1—C8	−35.9 (2)	C22—C15—C16—C21	0.1 (2)
C10—S1—C1—C8	78.3 (2)	S2—C15—C16—C21	−176.02 (16)
O3—S1—C1—C2	20.1 (2)	C22—C15—C16—C17	−180.0 (2)
O2—S1—C1—C2	149.75 (18)	S2—C15—C16—C17	3.9 (4)
C10—S1—C1—C2	−96.00 (19)	C21—C16—C17—C18	1.7 (3)
C8—C1—C2—C7	0.0 (2)	C15—C16—C17—C18	−178.2 (2)
S1—C1—C2—C7	175.20 (16)	C16—C17—C18—F2	−179.82 (19)
C8—C1—C2—C3	179.9 (2)	C16—C17—C18—C19	−0.8 (4)
S1—C1—C2—C3	−4.9 (4)	F2—C18—C19—C20	178.6 (2)
C7—C2—C3—C4	−1.0 (3)	C17—C18—C19—C20	−0.5 (4)
C1—C2—C3—C4	179.1 (2)	C18—C19—C20—C21	0.7 (3)
C2—C3—C4—F1	179.56 (18)	C19—C20—C21—O4	179.4 (2)
C2—C3—C4—C5	−0.2 (3)	C19—C20—C21—C16	0.3 (3)
F1—C4—C5—C6	−178.90 (19)	C22—O4—C21—C20	−177.9 (2)
C3—C4—C5—C6	0.9 (4)	C22—O4—C21—C16	1.3 (2)
C4—C5—C6—C7	−0.2 (3)	C17—C16—C21—C20	−1.6 (3)
C5—C6—C7—O1	−179.5 (2)	C15—C16—C21—C20	178.4 (2)
C5—C6—C7—C2	−1.1 (3)	C17—C16—C21—O4	179.21 (18)
C8—O1—C7—C6	178.1 (2)	C15—C16—C21—O4	−0.8 (2)
C8—O1—C7—C2	−0.5 (2)	C16—C15—C22—O4	0.7 (2)
C3—C2—C7—C6	1.8 (3)	S2—C15—C22—O4	176.73 (15)
C1—C2—C7—C6	−178.4 (2)	C16—C15—C22—C23	−178.4 (2)
C3—C2—C7—O1	−179.60 (17)	S2—C15—C22—C23	−2.4 (4)
C1—C2—C7—O1	0.3 (2)	C21—O4—C22—C15	−1.2 (2)
C7—O1—C8—C1	0.5 (2)	C21—O4—C22—C23	178.08 (18)
C7—O1—C8—C9	−177.92 (18)	O6—S2—C24—C28	−47.9 (2)
C2—C1—C8—O1	−0.3 (2)	O5—S2—C24—C28	−177.32 (17)
S1—C1—C8—O1	−175.62 (14)	C15—S2—C24—C28	68.63 (19)
C2—C1—C8—C9	177.7 (2)	O6—S2—C24—C25	70.9 (2)
S1—C1—C8—C9	2.4 (4)	O5—S2—C24—C25	−58.5 (2)
O3—S1—C10—C11	−50.09 (18)	C15—S2—C24—C25	−172.52 (18)
O2—S1—C10—C11	179.88 (16)	C28—C24—C25—C26	−29.6 (3)
C1—S1—C10—C11	64.59 (18)	S2—C24—C25—C26	−153.4 (2)
O3—S1—C10—C14	67.98 (17)	C24—C25—C26—C27B	23.9 (8)
O2—S1—C10—C14	−62.05 (17)	C24—C25—C26—C27A	45.8 (5)
C1—S1—C10—C14	−177.34 (15)	C25—C26—C27A—C28	−46.0 (9)
C14—C10—C11—C12	−17.1 (2)	C27B—C26—C27A—C28	74.3 (4)
S1—C10—C11—C12	103.3 (2)	C25—C26—C27B—C28	−8.7 (12)
C10—C11—C12—C13	36.0 (3)	C27A—C26—C27B—C28	−74.2 (4)
C11—C12—C13—C14	−41.4 (3)	C26—C27A—C28—C27B	−74.3 (4)
C12—C13—C14—C10	30.6 (3)	C26—C27A—C28—C24	27.8 (10)
C11—C10—C14—C13	−7.9 (2)	C26—C27B—C28—C27A	74.2 (4)
S1—C10—C14—C13	−129.75 (18)	C26—C27B—C28—C24	−10.5 (12)
O6—S2—C15—C22	17.4 (2)	C25—C24—C28—C27A	0.6 (7)
O5—S2—C15—C22	146.8 (2)	S2—C24—C28—C27A	122.7 (6)
C24—S2—C15—C22	−99.1 (2)	C25—C24—C28—C27B	25.2 (8)
O6—S2—C15—C16	−167.32 (18)	S2—C24—C28—C27B	147.2 (8)
O5—S2—C15—C16	−37.9 (2)		

### Hydrogen-bond geometry (Å, °)

**Table:**

*D*—H···*A*	*D*—H	H···*A*	*D*···*A*	*D*—H···*A*
C9—H9C···O6^i^	0.98	2.45	3.348 (3)	152
C19—H19···O6^ii^	0.95	2.58	3.469 (3)	157
C23—H23A···O2^iii^	0.98	2.50	3.318 (3)	141

Symmetry codes: (i) *x*−1, *y*−1, *z*; (ii) *x*−1, *y*, *z*; (iii) *x*+1, *y*, *z*.
